# Human Primary Odontoblast-like Cell Cultures—A Focused Review Regarding Cell Characterization

**DOI:** 10.3390/jcm11185296

**Published:** 2022-09-08

**Authors:** Christian Klein, Christian Meller, Edgar Schäfer

**Affiliations:** 1 HealthCare Center Meller Zahngesundheit Schlauzahn MVZ GmbH, D-71332 Waiblingen, Germany; 2 Department of Conservative Dentistry, Periodontology and Endodontology, University Centre of Dentistry, Oral Medicine and Maxillofacial Surgery, University Hospital Tübingen, D-72076 Tübingen, Germany; 3 Central Interdisciplinary Ambulance in the School of Dentistry, University of Münster, D-48149 Münster, Germany

**Keywords:** cell culture, characterization, odontoblast-like, osteoblast-like, stem cell

## Abstract

Cell cultures can provide useful in vitro models. Since odontoblasts are postmitotic cells, they cannot be expanded in cell cultures. Due to their extension into the dentin, injuries are inevitable during isolation. Therefore, “odontoblast-like” cell culture models have been established. Nowadays, there is no accepted definition of odontoblast-like cell cultures, i.e., isolation, induction, and characterization of cells are not standardized. Furthermore, no quality-control procedures are defined yet. Thus, the aim of this review was to evaluate both the methods used for establishment of cell cultures and the validity of molecular methods used for their characterization. An electronic search was performed in February 2022 using the Medline, Scopus, and Web of Science database identifying publications that used human primary odontoblast-like cell cultures as models and were published between 2016 and 2022. Data related to (I) cell culture conditions, (II) stem cell screening, (III) induction media, (IV) mineralization, and (V) cell characterization were analyzed. The included publications were not able to confirm an odontoblast-like nature of their cell cultures. For their characterization, not only a similarity to dentin but also a distinction from bone must be demonstrated. This is challenging, due to the developmental and evolutionary proximity of these two tissue types.

## 1. Introduction

The advantage of cell cultures is that experiments can be conducted under defined conditions, ensuring the comparability of results and repeatability of experiments [1]. Nevertheless, cell cultures are model systems that, like all models, are designed to provide experimental access to a complex reality [2]. The simplification of reality inherent in a model is therefore both a necessity and a risk. The risk lies in the uncritical transfer of experimental results from the model to reality without verifying whether this is due to the simplifications made.

In cell culture models, another risk arises from an insufficient characterization of the cell cultures. For primary cell cultures, isolation from the original tissue usually results in a heterogeneous cell population. This can be affected additionally by cross-contamination with other cultures present in the laboratory. Finally, induction and differentiation of stem cells is also a potential source of error [3]. Consequently, knowledge of the origin and structure of the source tissue and its cell populations is essential for the characterization of any cell culture.

The term “characterization” includes not only the identification of cell lines and the detection of their distinctive features in relation to the tissue of origin but also authentication (i.e., exclusion of misidentification or cross-contamination), the conformation of the species of origin, the identification of signs of malignancy, phenotype variation, and genetic instability [4]. Nevertheless, identification of primary odontoblast-like cell cultures by the detection of distinguishing features is the usual procedure. The perception of what distinctive features are has changed over time with the progress of laboratory technology and the associated increase in knowledge. So far, there is no accepted definition of primary odontoblast-like cell cultures, i.e., isolation, induction, and characterization of cells have not been standardized. Furthermore, no quality-control procedures have been defined yet. Notwithstanding this lack of clarity and since mature odontoblasts are postmitotic cells that cannot be expanded in cell cultures [5,6], putative odontoblast-like cell cultures have been used as models to investigate cytotoxic [7], material [7,8,9], pharmacological [10,11], and physiological [12,13,14,15,16] research questions in dentistry.

The exposed considerations motivated the aim of the present focused review, which was to evaluate the methods used to establish cell cultures and the validity of the molecular methods used to characterize them. Based on an electronic search, recent publications were identified in which human primary odontoblast-like cell cultures were applied as an in vitro model.

## 2. Methods

### 2.1. Search Strategy

This analysis was reported following the Preferred Reporting Items for Systematic Review and Meta-Analyses statement (PRISMA) [17]. An electronic search was performed accessing the Medline database of the National Library of Medicine (NLM), the Scopus database (Elsevier B.V., Amsterdam, Netherlands), and the Web of Science database (Clarivate Plc, Philadelphia, PA, USA) in February 2022. The search term used was “human odontoblast-like cells” and the publication period chosen was 2016–2022. No Boolean operators were applied. The bibliography and the list of similar articles suggested by PubMed for the retrieved articles were searched for possible additional relevant publications.

### 2.2. Inclusion and Exclusion Criteria

The inclusion and exclusion criteria are summarized in Table 1. Only original papers that were written in English for which access to the full text was available and that were not published before 2016 were considered so as to ensure contemporary methods. Furthermore, only articles that had used human primary odontoblast-like cell cultures were of interest. Articles describing odontoblast-like cell cultures from animals, organ cultures [18], immortalized cell lines, or spheroid cell cultures were excluded.

### 2.3. Data Extraction

Articles included in this analysis were evaluated regarding the cell culture technique used, the identification and induction of mesenchymal stem cells, the mineralization of the induced cell cultures, and the methods applied to characterize the induced cell cultures, if the information provided by the authors allowed it. To assess the cell culture technology, the source and processing of the original tissue, the cell culture medium and its additives, and references to previous published work were reviewed.

Regarding mesenchymal stem cells, it was assessed whether the expression of stem cell markers was examined, and if so, the methodology used. Furthermore, the formulation of the induction medium and the duration of its application were documented. Finally, the passages were recorded, with which the final experiments were conducted.

To assess the mineralization of cell cultures, it was examined whether the expression of alkaline phosphates (ALPL) was assessed, whether their activity was determined, and whether the mineralization was visualized or quantified by staining (i.e., alizarin red or von Kossa). If immunohistochemistry or Western blot (i.e., protein identification methods) was chosen for characterization, the information provided by the authors and manufacturers on the antibodies used was evaluated.

In cases where gene expression was used to characterize cell culture, the published sequence of the forward and reverse primers as well as the product size of the amplicon were documented. To assess the quality of the published primer sequences, a gene search was performed in the Reference Sequence database (RefSeq), built by the National Center of Biotechnology Information (NCBI; Bethesda, SA). The tool “Find” in this sequence was used to identify the amplicon within the sequence. The start and end positions, product size, and primer sequences were recorded for the identified amplicons. For the DSPP gene, the amplified exon was also identified.

Finally, published data on RT-(q)PCR parameters were reviewed following the MIQE (minimum information for publication of quantitative real-time PCR experiments) guidelines [19].

## 3. Results

### 3.1. Literature Search and Screening Process

**Figure 1:**
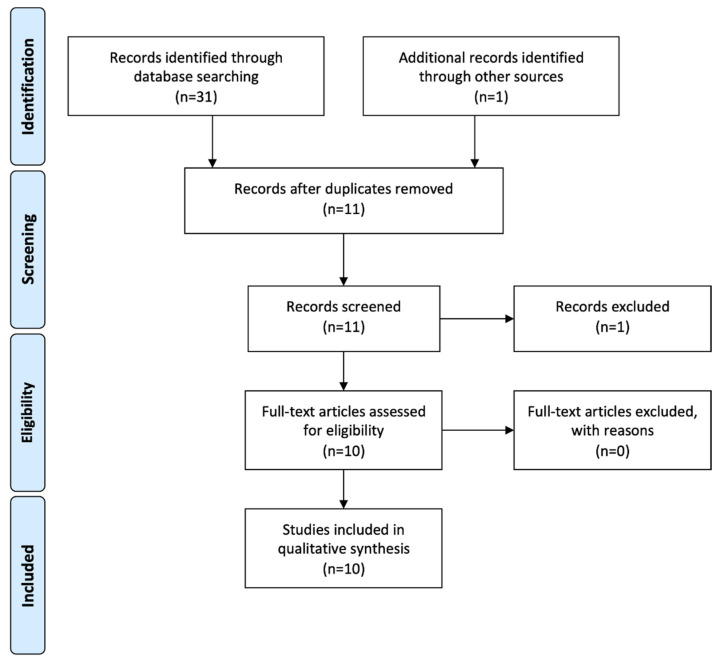
PRISMA flowchart of literature search and screening process.

The electronic literature search yielded a total of 31 articles and the hand search one further article. After duplicates were removed, eleven articles remained. Of these, one was excluded after screening the abstract [20]. Ten articles fulfilled the inclusion criteria and were subsequently reviewed [7,8,9,10,11,12,13,14,15,16]. The flowchart summarizing the screening process is presented in Figure 1.

### 3.2. Cell Culture

Sun et al. [7] were the only ones not mentioning the origin of their cell cultures. All others specified third molars as the source. The maximum age of the donors was given as either 18 [10,12,14,16], 25 [7,8,13] or 35 years [11]. Only one publication vaguely reported on “young patients” [9]. Mechanical methods were predominantly used to disrupt the original tissue. Baldion et al. [8,9], Latorre et al. [10], and Feng et al. [15] were the only ones to use collagenase I plus dispase digestion, Baldion et al. overnight, Latorre et al. for 16 h and Feng et al. for 20 min. Further details on cell culture conditions are given in Table 2.

### 3.3. Mesenchymal Stem Cell Screening

Only Latorre et al. [10] described screening for mesenchymal stem cells in their article. Baldion et al. described a screening in their preliminary work [21]. Using flow cytometry, it was shown that the primary cells prior to induction had the marker profile of mesenchymal stem cells (CD14-, CD19-, CD20-, CD34-, CD 45-, CD73+, CD90+, CD105+). The data in both publications of Baldion et al. [8,9] suggest that screening was performed as a general quality-assurance measure. No other publications described screening for mesenchymal stem cells.

### 3.4. Induction Medium

Usually, dexamethasone, β-glycerophosphate and ascorbic acid were supplemented by the basic cell culture medium for stem cell induction of mineralizing tissue. Liu et al. [14], Wen et al. [12], and Sun et al. [7] did not add dexamethasone, and Liu et al. [14] and Wen et al. [12] also did not add ascorbic acid. Baldion et al. [8,9] and Latorre et al. [10] were the only researchers that additionally added TGF-β1 to the basic cell culture medium. Only six publications [7,8,9,10,15,16] provided information on the application period of the induction medium, which varied from 2 to 28 days. Four publications did not specify this.

Which passages of the cell cultures were used for the experiments [8,9,10,11]. Another publication remained undefined with the statement that cell cultures were used from the third passage onwards, but the upper limit was not given [12]. The remaining publications described the application of the second to sixth passages. Further details on the induction medium, its duration of application, and the passages used for the experiments are given in Table 3.

### 3.5. Mineralization

Sun et al. determined the expression of alkaline phosphatase by semiquantitative RT-PCR [7]. The published primers were found within the NCBI sequence NM_000478. Sabandal et al. quantified alkaline phosphatase activity and calcium phosphate formed using commercial assays [11]. However, this was not done to demonstrate the differentiation of the cell cultures, but to investigate the effect of simvastatin on their mineralization. Liu et al. demonstrated nodule formation using alizarin red staining [16]. Baldion et al. described the quantification of alkaline phosphatase by RT-qPCR in their previous work [21], but the application of this method [8,9] remains unclear. The published primers were found in the NCBI sequence NM_000478, although the amplicon size of 458 bp was smaller than the described size of 476 bp. In all other publications reviewed, it was not stated whether the mineralization of the cell cultures had been investigated. Further details can be found in Table 4.

### 3.6. Characterization

To assess the characterization of the cell cultures as odontoblast-like, the information provided in the included studies was evaluated with regard to the expression of DSPP, DMP1, and nestin. This was done both at the RNA level by RT-PCR and at the protein level by immunohistochemistry and Western blotting. This resulted in a wide range of methods used. Feng et al. [15] reported all three methods for all three genes assessed, whereas Meng et al. [13] reported none. DSPP was the gene most often used for characterization (nine out of ten publications). DMP1 and nestin were each used by only four research groups. Sun et al. were the only research group to use bone sialoprotein (IBSP) as a marker with RT-qPCR [7]. An overview of the cell culture characterization methods used in the publications is shown in Table 5.

### 3.7. Immunohistochemistry and Western Blotting

DSPP was identified at the protein level using antibodies from Abcam Plc (Cambridge, UK) by Baldion et al. [8,9], Bioss Inc. (Boston, USA) by Liu et al. [16], and Santa Cruz Biotechnology Inc. (Santa Cruz, USA) by Feng et al. [15], Liu et al. [14], and Wen et al. [12]. Abcam Plc and Bioss Inc. do not specify in their data sheet against which part of the protein the antibody is targeting, whereas Baldion et al. [21] described the Abcam plc antibody as an anti-DSP antibody. In contrary, Santa Cruz Biotechnology Inc. states in its data sheet that there is no cross-reactivity with DSP, i.e., it targets DPP. Further details of the anti-DMP1 and anti-nestin antibodies used are shown in Table 5.

### 3.8. RT-PCR

To identify DSPP expression with RT-PCR, three research groups used quantitative PCR [8,9,12,15], one PCR in combination with gel electrophoresis [7], and one solely non-quantitative PCR [11]. The published primer sequences were compared with the NCBI Reference Sequence NG_011595.1 of the DSPP gene. Two primer sequences could not be matched at all [12,15], two amplified a part of exon 4 [7,11], and one amplified a part of exon 5 [8,9]. Further details are given in Table 6.

DMP1 primer sequences published by Baldion et al. [8] and Feng et al. [15] were compared with NCBI Reference Sequences NM_004407.4, NM_001079911.3, XM_011531705.2, and XM_011531706.2. No matches could be found. The same was true when comparing the primer sequences for nestin published by Feng et al. [15] and Wen et al. [12] with NCBI Reference Sequences NG_012300.1 and NM_006617.2.

### 3.9. RT-(q)PCR Parameters

All publications reported the primer sequences of the assays; however, Baldion et al. [8,9] were the only ones to report an accession number. None of the research groups provided information on analyzing of the isolated RNA for integrity (e.g., gel electrophoresis), nor did they describe control reactions to detect DNA or cross-contamination during PCR. Baldion et al. included PCR efficiency when calculating gene expression levels using the LinRegPCR software [28] and Wen et al. used the REST 2005 software [29]. Further details of the PCR parameters applied are shown in Table 7.

## 4. Discussion

A literature review was conducted to identify studies that used human primary odontoblast-like cell cultures as an in vitro model. Rodents are often used as experimental animals or sources [30,31] for the establishment of cell cultures. However, in contrast to humans, they have continuously growing teeth, so the comparability of results is limited [32]. In addition, species differences exist in the chemical composition of the organic matrix [33]. In contrast, human third molars are an easily available source. For this reason, only human cell culture models were considered. Immortalized cell lines were not included since the characterization must be undertaken only once. Therefore, more expensive and time-consuming methods (e.g., microarray technique) can be applied. Furthermore, immortalized cell lines were obtained from a single patient. The biological variance is not replicated by such cell lines. After all, the metabolism of the cells may change because of the immortalization. Since the minimal criteria for defining multipotent mesenchymal stem cells (MSCs) by the International Society for Clinical Therapy [34] include adherence to plastic, spheroid cell cultures were excluded. Finally, only studies published after 2015 were included. This was to ensure that current methods were used for characterization. The publication by Yamamoto et al. [35] was chosen as a limiting factor because they demonstrated two splice variants for the DSPP gene. This must be considered when evaluating antibodies and primers targeting this gene.

### 4.1. Cell Characterization

In the included studies, the characterization of cell cultures as odontoblast-like was implemented through the identification of distinct features. To assess the validity of the characterization, both the concept and its methodological implementation must be evaluated. The best identification is useless if aspecific features are chosen, and the most specific features are worthless if their identification is inadequate. Nevertheless, methodological shortcomings are less severe than conceptual ones because these can be corrected.

In 2011, Goldberg et al. observed [36] that using a single master regulatory molecule for identification is naïve. This applies not only for the regulatory processes leading to the differentiation of dental papilla cells into odontoblasts but also for the proteins of the extracellular matrix (ECM) secreted by these odontoblasts, which control the mineralization of dentin. Nevertheless, in one publication [11], purely the qualitative demonstration of DSPP expression was considered sufficient to characterize the cell cultures as odontoblast-like.

When applying qPCR with the comparative ΔΔCT method [37], a cell or tissue type must be used as reference. Undifferentiated pulp cell cultures (referred to as human dental pulp cells [15], human dental pulp stem cells [21], or human cultured pulp fibroblasts [38]) were taken as a reference for the human odontoblast-like cell cultures. Of the included publications, only one reported the measured values [15]. Thereby, DSPP was expressed threefold on the RNA level and 30-fold on the protein level and DMP1 was expressed 3.5-fold and tenfold, respectively. For the RNA level, Baldion et al. [21] specified in their preliminary work the expression for DSPP as 5- to 32-fold and for DMP1 as 63- to 90-fold, depending on the induction period. Nevertheless, the question arises whether an undifferentiated cell culture is the appropriate benchmark. Klein et al. [39] isolated total RNA from the odontoblast layers of three caries-free impacted wisdom teeth with open apices of the same male donor (15 years old) and from the third passage of uninduced pulp-derived cell cultures obtained from the same teeth, cultivated with DMEM supplemented with 8% FCS. RT-qPCR was performed utilizing RT2 Profiler PCR arrays (Qiagen GmbH, Hilden, Germany) and normalization was achieved by geometric averaging the data of three housekeeping genes [40]. Here, in the odontoblast layer, DSPP was expressed 380,000-fold and DMP1 19,000-fold relative to the undifferentiated cell culture. Taking these numbers into account, the question arises as to how similar a cell culture must be to the odontoblast layer to merit the characterization “odontoblast-like” and to be suitable as a reliable model.

The expression of putative odontoblast-specific marker genes or proteins in combination with the fact that the cell cultures were obtained from pulp tissue was considered sufficient evidence for the establishment of odontoblast-like cell cultures. With the demonstration of mesenchymal stem cells in the pulp (DPSCs) by Grontos et al. [41], this argument is no longer valid. The International Society for Cellular Therapy lists multipotent differentiation into osteoblasts, adipocytes, and chondroblasts, in addition to adherent growth on plastic and expression of specific surface antigens, as minimal criteria for the identification of these cells [34], and one of the studies reviewed demonstrated this for the cell cultures used [10]. Osteogenic differentiation of DPSCs was published to occur both in vitro [10,42,43] and in vivo [44]. The differences in the induction media were minimal. All studies used β-glycerophosphate as an osteogenic [10,42,45,46,47,48] or odontogenic differentiation supplement and combined it with dexamethasone and/or ascorbic acid (Table 3). Therefore, the additional question arises whether and how osteoblast-like and odontoblast-like cell cultures can be distinguished in vitro, especially when derived from the same DPSC population.

Both bone and dentin are mineralized tissue and their inorganic component consists of hydroxyapatite. However, biomineralization in vertebrates is a process mediated by an extracellular organic matrix [49]. In dentin, this ECM is known as predentin and secreted by odontoblasts; in bone, osteoblasts secrete the ECM osteoid. In contrast to enamel, where the ECM is (transiently) composed predominantly of amelogenin (AMEL). In bone and dentin, it is made up of approximately 90% type I collagen [36,50]. Nevertheless, the initiation and control of matrix mineralization is mediated by noncollagenous proteins (NCPs). In addition to proteoglycans and unphosphorylated proteins (bone gamma-carboxyglutamate protein (BGLAP), secreted protein acidic and cysteine rich (SPARC)), these include a group of acidic proteins, which belong to the secretory calcium-binding phosphoprotein (SCPP) family [51]. In tetrapods, proteins of this family regulate extracellular calcium phosphate concentrations and matrix mineralization and comprise enamel SCPPs (ameloblastin (ABMN), enamelin (ENAM), amelogenin (AMEL)), dentin/bone SCPPs, also known as small integrin-binding ligand N-linked glycoproteins (SIBLINGs) [52,53], (dentine sialophosphoprotein (DSPP), dentine matrix acidic protein 1 (DMP1), bone sialoprotein (IBSP), matrix extracellular phosphoglycoprotein (MEPE), and osteopontin (SPP1)), eggshell matrix proteins in birds (ovocleidin 116 (OC116)), and milk caseins (α_s1_-casein (CSN1S1), α_s2_-casein (CSN1s2), and β-casein (CSN2)) and salivary proteins (Statherin (STAHT), proline-rich proteins (PROL), and histatin (HTN)) in mammals [54,55]. Except for AMEL, which is localized on X and Y chromosomes, all SCPPs in humans are encoded on chromosome 4, the two enamel SCPPs on 4q13 and the SIBLINGs on 4q21. Furthermore, the SIBLINGs share a similar exon–intron organization, one or more consensus sequences for phosphorylation by casein kinase II, and an RGD integrin-binding site. It has therefore been suggested that they evolved from a common ancestor by gene duplication [51,52].

The expression of SIBLINGs has been detected in both bone and dentin, which, together with organization on human chromosome 4q21 and an evolution by gene duplication, suggests a close evolutionary relationship between these two tissue types [56]. Thus, the differences between the tissue arise from the concentrations and posttranslational modifications of these proteins [57,58,59]. Hall and Witten [60] stated that dental and skeletal tissue represent a continuum from a developmental and evolutionary perspective, with dentin and bone representing the two edges. As such, the respective expression is influenced by the environment. Therefore, due to the similarity of odontogenic and osteogenic induction media, it must be doubted that either edge is fully reached with respect to primary pulp-derived cell cultures. This is also supported by the fact that transplanting stem cells into an experimental animal, as Gronthos et al. [41] did in 2000, is still considered the gold standard to demonstrate stem cell differentiation. Since the differences arise among others from the content of SIBLINGs in the respective ECMs, the expression must be analyzed quantitatively (i.e., RT-qPCR and Western blotting). For instance, DSPP in dentin is the second most abundant component of the ECM after type I collagen. In bone, it is found at a level of 1/400 of that in dentin [61]. Given this, is a threefold upregulation at the RNA level and a 30-fold at the protein level comparable with the undifferentiated cell culture [15] evidence for an odontoblast- or for an osteoblast-like cell culture? However, this remains an open question, which in turn leads to the question of the appropriate benchmark discussed above.

Since the relative ratio of SIBLINGs in dental and skeletal tissue determines their formation, this should also be a feature to include in the characterization. For the values cited above, in one study [15] the ratios of DSPP and DMP1 were approximately the same with three and 3.5-fold expression, respectively. In the other study [21], DSPP was significantly less expressed at 5–32-fold than DMP1 at 63–90-fold. Therefore, the significantly lower DSPP expression in the latter study would suggest a more osteoblast-like cell culture, as DSPP is more weakly expressed in bone [61]. The studies include in this review did not quantify the expression of all SIBLINGs, and of those that were quantified, the values were reported in only one study.

In addition to the two SIBLINGs DSPP and DMP1, nestin (neural stem cell protein) was used as a marker for the odontoblast-like nature of cell cultures in the studies reviewed (Table 5). Nestin, as an intermediate filament, is part of the cytoskeleton of cells and has been identified as a marker for neural stem cells [62]. In addition, it was detected in several cell and tissue types during embryogenesis (e.g., skeletal [62] and cardiac [63] muscles, testicular cells [64], and renal progenitors [65]) and in adults (e.g., pancreatic islet cells [66], retina [67], myocardium [68], renal podocytes [65], and mammary glands [69]). Furthermore, it was identified in DPSCs due to their neural crest origin [70]. Therefore, nestin expression is not specific to neural tissue and is expressed under both healthy and pathological conditions [32,71]. For teeth, About et al. [32] described the expression of nestin beginning with the bell stage in odontoblasts, where it is downregulated at maturity, but also in the pulp tissue. Furthermore, Wong et al. [72] demonstrated that nestin expression did not change during osteogenic differentiation of equine, canine, or human bone marrow-derived MSCs. Therefore, nestin seems not to be a suitable marker for any distinction between pulp tissue, odontoblast-like and osteoblast-like cell cultures.

Finally, most included studies reviewed failed to provide evidence of ALPL expression or mineralization (i.e. von Kossa [73] or Alizarin red [74] staining) of their cell cultures (Table 4). ALPL expression and calcium phosphate deposition are necessary but not sufficient indicators of an odontoblast-like or osteoblast-like cell type [75]. Since DPSCs are a very heterogeneous population with many subgroups, only two-thirds of which are capable of forming dentin in vivo [76], mineralization detection should be performed at least for the purpose of quality assurance.

### 4.2. Strengths and Limitations of This Review

The strengths of this review are that the methods used for establishment of cell cultures and the validity of molecular methods used for characterization of human odontoblast-like cell cultures were analyzed in terms of several aspects. To the best of the authors’ knowledge, similar analyses have not been performed before. Moreover, the literature search process and the following data extraction were performed by at least two independent reviewers.

On the other hand, limitations of this review are twofold. Firstly, one or two additional studies might have been found with more elaborate search strategies (i.e., the use of further search terms or Boolean operators). Nonetheless, the ten studies reviewed here ensure a representative sample. Secondly, the fact that the language of the included studies was restricted to English may represent a language bias, as this excludes research in this area that was published in other languages.

## 5. Conclusions and Recommendations

The included studies were neither methodologically nor conceptually able to confirm an odontoblast-like nature of cell cultures. This was due to the fact that the distinction was thought to be only in the direction of pulp tissue or undifferentiated cell culture. However, the source is multipotent mesenchymal stem cells from the pulp, and the cell culture supplements used for differentiation are recommended for both osteogenic and odontogenic differentiation. Therefore, for the characterization of cell cultures as odontoblast-like, not only a similarity to dentin but also a distinctness from bone must be demonstrated. This verification is much more challenging due to the developmental and evolutionary proximity of these two tissues. Since findings obtained with a model can only be meaningfully compared and transferred to clinical reality if the model has been standardized, the following recommendations are made:

- When extracting third molars, the apex should still be open and root growth should not yet be completed, regardless of the age of the donor. This ensures that the odontoblast layer still forms primary dentin [77] and that a significant expression profile can be identified.
- Total RNA for reference purposes should be extracted from both the odontoblast layer and the pulp tissue of each tooth from which a cell culture is established.
- RT-qPCR of all SIBLINGS, BGLAP, SPARC, ALPL and COL1A1 as target genes should be performed not only for the differentiated and undifferentiated cell cultures but also for the odontoblast layer and pulp tissue of the respective tooth and reported according to MIQE guidelines [19].
- For undifferentiated cell cultures, at least the expression of the surface antigens should be demonstrated from the criteria for mesenchymal stem cells of the International Society for Cellular Therapy [34].
- The mineralization of the differentiated cell cultures should be documented by a suitable staining method.
- The number of biological replicates (i.e., donors) should be reported unambiguously.
- For better reader comprehension and to ensure repeatability of the experiments, the complete methodology of the cell culture and its characterization, including the quality-assurance measures, should be given separately and as a summary for each cell culture/donor, without references to other publications and their results.
- To ensure the comparability of results, international societies (e.g., European Society of Endodontology, American Association of Endodontists) should define minimum standards for the establishment of odontoblast-like cell cultures and their characterization.

Nevertheless, future studies will have to address the question of whether clearly distinguishable odontoblast- or osteoblast-like cell culture models can be established, e.g., by optimizing the induction media or the technique for stem cell isolation.

## Figures and Tables

**Table 1:** Inclusion and exclusion criteria.

Criterion	Inclusion	Exclusion
Organism	human	animals
Cell culture	primary cell cultures	organ cultures immortalized cell lines spheroid cell cultures
Publication date	2016–2022	published before 2016
Access	Full-text access	no full-text access
Language	English	others
Article type	original article	review, data brief, etc.
Review	journals with peer review	journals without peer review

**Table 2:** Overview of cell culture sources, cell culture media, and their supplements. Information in *italics and blue* are additions that were made based on the cited preliminary work, but were missing in the materials and methods sections of the included articles.

Publication	Cited Publication	Source	Digestion Process	Medium	FBS	Antibiotics	Antifungal Configuration
Baldion et al. (2021) [8]	Baldion et al. (2018) [21]	third molars (14–18 years)	*3 mg/mL collagenase I + 4 mg/mL dispase (overnight)*	DMEM	10%	penicillin + streptomycin	none
Feng et al. (2021) [15]	Couble et al. (2000) [22]/Zhang et al. (2015) [23]	third molars (17(18)–25 years)	*3 mg/mL collagenase I (20 min)*	DMEM	10%	100 µg/mL penicillin + 100 mg/mL streptomycin	none
Baldion et al. (2021) [9]	Baldion et al. (2018) [21]	third molars (young patients)	*3 mg/mL collagenase I + 4 mg/mL dispase (overnight)*	DMEM (lg)	10%	100 µg/mL penicillin + 100 µg/mL streptomycin	none
Liu et al. (2021) [16]	About et al. (2000) [24]	third molars (age not stated)	no digestion	DMEM	10%	100 U/mL penicillin + 100 mg/mL streptomycin	none
Sabandal et al. (2020) [11]	Jung et al. (2018) [25]/Sielker et al. (2020) [26]	third molars (18–35 years)	no digestion	DMEM (lg)	10%	10.000 U/mL penicillin + 10.000 g/mL streptomycin	250 mg/mL amphotericin B
Liu et al. (2020) [14]	About et al. (2000) [24]/Lee et al. (2016) [27]	third molars (16–18 years)	*no digestion*	MEM	10%	100 U/mL penicillin + 100 mg/mL streptomycin	0.25 mg/mL amphotericin B
Latorre et al. (2020) [10]	Baldion et al. (2018) [21]	third molars (14–18 years)	3 mg/mL collagenase I + 4 mg/mL dispase (16 h)	DMEM (lg)	10%	100 U/mL penicillin + 100 µg/mL streptomycin	none
Meng et al. (2019) [13]	Couble et al. (2000) [22]	third molars (17–25 years)	*no digestion*	DMEM	10%	100 µg/mL penicillin + 100 mg/mL streptomycin	none
Wen et al. (2017) [12]	About et al. (2000) [24]/Lee et al. (2016) [27]	third molars (16–18 years)	*no digestion*	MEM	10%	100 U/mL penicillin + 100 mg/mL streptomycin	0.25 mg/mL amphotericin B
Sun et al. (2018) [7]	-	teeth not specified (12–25 years)	no digestion	DMEM	10%	0.2% penicillin/streptomycin	none

Abbreviations: (D)MEM (lg) = (Dulbecco’s) modified Eagle medium (low glucose); FBS = fetal bovine serum.

**Table 3:** Overview of the data on the formulation of the induction media, their duration of application, and the passages used for the experiments. The concentrations of dexamethasone and ascorbic acid reported by Sabandal et al. [11] were converted to the units used by the other research groups and are added in parentheses for easier comparison. Information in *italics and blue* are additions that were made based on the cited preliminary work, but were missing in the materials and methods sections of the included articles, in addition to. Reproduced with permission of Sabandal et al., Materials; published by MDPI, 2020.

Publication	Cited Publication	Dexamethasone	β-glycerophosphate	Ascorbic Acid	TGF-β1	Application Period	Passages Used
Baldion et al. (2021) [8]	Baldion et al. (2018) [21]	0.1 µM	5 mM	50 µg/mL	10 ng/mL	21 days	not stated
Feng et al. (2021) [15]	Couble et al. (2000) [22]/Zhang et al. (2015) [23]	0.1 µM	10 mM	50 µg/mL	none	*2–14 days*	2nd–3rd
Baldion et al. (2021) [9]	Baldion et al. (2018) [21]	0.1 µM	5 mM	50 µg/mL	10 ng/mL	21 days	not stated
Liu et al. (2021) [16]	About et al. (2000) [24]	10^-7^ mM (0.1 nM)	10 mM	50 µg/mL	none	21 days	3rd–6th
Sabandal et al. (2020) [11]	Jung et al. (2018) [25]/Sielker et al. (2020) [26]	16 ng/mL (0.04 µM)	10 mM	1.4 mM (2465 µg/mL)	none	not stated	not stated
Liu et al. (2020) [14]	About et al. (2000) [24]/Lee et al. (2016) [27]	none	2 mM	none	none	not stated	4th–6th
Latorre et al. (2020) [10]	Baldion et al. (2018) [21]	0.1 µM	*5 mM*	50 µg/mL	10 ng/mL	7, 14 and 21 days	not stated
Meng et al. (2019) [13]	Couble et al. (2000) [22]	0.1 µM	10 mM	50 µg/mL	none	not stated	2nd–3rd
Wen et al. (2017) [12]	About et al. (2000) [24]/Lee et al. (2016) [27]	none	2 mM	none	none	not stated	3rd
Sun et al. (2018) [7]	-	none	10 mM	5 mg/mL	none	21–28 days	3rd–5th

Abbreviation: TGF-β1 = transforming growth factor β1.

**Table 4:** Overview of techniques used to visualize mineralization. Information in *italics and blue* are additions that were made based on the cited preliminary work but were missing in the materials and methods sections of the included articles.

Publication	Cited Publication	PCR (ALPL)	Histology (ALPL)	Wetern Blot (ALPL)	Activity (ALPL)	Von Kossa Staining	Alizarin Red Staining
Baldion et al. (2021) [8]	Baldion et al. (2018) [21]	*qPCR (primers specified)*			*Alkaline Phosphatase Assay Kit (Abcam)*	*yes*	*yes*
Feng et al. (2021) [15]	Couble et al. (2000) [22]/Zhang et al. (2015) [23]						
Baldion et al. (2021) [9]	Baldion et al. (2018) [21]				*Alkaline Phosphatase Assay Kit (Abcam)*	*yes*	*yes*
Liu et al. (2021) [16]	About et al. (2000) [24]						yes
Sabandal et al. (2020) [11]	Jung et al. (2018) [25]/Sielker et al. (2020) [26]				*Alkaline Phosphatase Assay Kit (Abcam)*		Alizarin Red S Staining Quantification Assay (ScienCell)
Liu et al. (2020) [14]	About et al. (2000) [24]/Lee et al. (2016) [27]						
Latorre et al. (2020) [10]	Baldion et al. (2018) [21]						*yes*
Meng et al. (2019) [13]	Couble et al. (2000) [22]						
Wen et al. (2017) [12]	About et al. (2000) [24]/Lee et al. (2016) [27]						
Sun et al. (2018) [7]	-	PCR + electrophoresis (primers specified)					

Abbreviations: ALPL = alkaline phosphatase, biomineralization associated (tissue non-specific alkaline phosphatase); (q)PCR = (quantitative) polymerase chain reaction.

**Table 5:** Overview of the methods and genes used for cell culture characterization. Information in *italics and blue* are additions that were made based on the cited preliminary work or manufacturer’s data but were missing in the materials and methods sections of the included articles.

Publication	Cited Publication	PCR (DSPP)	Histology (DSPP)	Western Blot (DSPP)	PCR (DMP1)	Histology (DMP1)	Western Blot (DMP1)	PCR (Nestin)	Histology (Nestin)	Western Blot (Nestin)
Baldion et al. (2021) [8]	Baldion et al. (2018) [21]	*qPCR (primers reported)*	*rabbit polyclonal anti-DSP antibody (Abcam)*		qPCR (primers reported)	rabbit polyclonal antibody (Sigma-Aldrich)				
Feng et al. (2021) [15]	Couble et al. (2000) [22]/Zhang et al. (2015) [23]	qPCR (primers reported)	*mouse polyclonal* antibody (Santa Cruz)	*mouse polyclonal* antibody (Santa Cruz)	qPCR (primers reported)	*rabbit monoclonal* antibody (Abcam)	*rabbit monoclonal* antibody (Abcam)		*rabbit monoclonal* antibody (Abcam)	*rabbit monoclonal* antibody (Abcam)
Baldion et al. (2021) [9]	Baldion et al. (2018) [21]	*qPCR (primers reported)*	*rabbit polyclonal anti-DSP antibody (Abcam)*		*qPCR (primers reported)*	*rabbit polyclonal antibody (Sigma Aldrich)*				
Liu et al. (2021) [16]	About et al. (2000) [24]		rabit polyclonal anti-DSPP antibody (Bioss)	rabit polyclonal anti-DSPP antibody (Bioss)					mouse polyclonal antibody (Proteintech Group)	mouse polyclonal antibody (Proteintech Group)
Sabandal et al. (2020) [11]	Jung et al. (2018) [25]/Sielker et al. (2020) [26]	PCR (primers reported)								
Liu et al. (2020) [14]	About et al. (2000) [24]/Lee et al. (2016) [27]		mouse polyclonal antibody (Santa Cruz)						rabbit polyclonal antibody (Proteintech Group)	
Latorre et al. (2020) [10]	Baldion et al. (2018) [21]		rabbit polyclonal anti-DSP antibody (Abcam)			rabbit polyclonal antibody (Sigma-Aldrich)				
Meng et al. (2019) [13]	Couble et al. (2000) [22]									
Wen et al. (2017) [12]	About et al. (2000) [24]/Lee et al. (2016) [27]	qPCR (primers reported)	mouse polyclonal antibody (Santa Cruz)					qPCR (primers reported)	rabbit polyclonal antibody (Proteintech Group)	
Sun et al. (2018) [7]	-	PCR + electrophoresis (primers reported)								

Abbreviations: DMP1 = dentin matrix acidic phosphoprotein 1; DSP = dentin sialoprotein; DSPP = dentin sialophosphoprotein; (q)PCR = (quantitative) polymerase chain reaction.

**Table 6:** The table summarizes the published data on the RT-PCR of the DSPP gene and the results found by the Reference Sequence (RefSeq) database search. In the “Sequence found (reverse primer)” column, the sense sequence of the reverse primer was also given in parentheses to facilitate comparison with the NCBI Reference Sequence. Information in *italics and blue* are additions that were made based on the cited preliminary work but were missing in the materials and methods sections of the included articles.

Publication	Cited Publication	PCR	Forward Primer Published	Reverse Primer Published	Product Size Published	Sequence Found (Forward Primer)	Start Position	Sequence Found (Reverse-Primer)	End Position	Product Size Found	Amplicon Position
Baldion et al. (2021) [8,9]	Baldion et al. (2018) [21]	*qPCR*	*GGCAGTGCATCAAAAGGAGC*	*TGCTGTCACTGTCACTGCTG*	not stated	ggcagtgactcaaaaggagc	10,644	tgctgtcactgtcactgctg (cagcagtgacagtgacagca)	10,848	205 bp	Exon 5
Feng et al. (2021) [15]	Couble et al. (2000) [22]/Zhang et al. (2015) [23]	qPCR	GGCTGAGATGAGGCAAAAAG	ACCAACTCGGTACAGGATGC	not stated	not found					
Sabandal et al. (2020) [11]	Jung et al. (2018) [25]/Sielker et al. (2020) [26]	PCR	GTCGCTGTTGTCCAAGAAGA	ATCCTCATCTGCTCCATTCC	239 bp	gtcgctgttgtccaagaaga	9184	atcctcatctgctccattcc (ggaatggagcagatgaggat)	9420	237 bp	Exon 4
Wen et al. (2017) [12]	About et al. (2000) [24]/Lee et al. (2016) [27]	qPCR	CAGTACAGGATGAGTTAAATGCAAGTG	CCATCCCTTCTCCCTTGTGACC	118 bp	not found					
Sun et al. (2018) [7]		PCR + electrophoresis	GGTGTCCTGGTGCATGAAGGT	CCTCGTCTTCATCCTCATCTG	601 bp	ggtgtcctggtgcatgaaggt	8830	cctcgtcttcatcctcatctg (cagatgaggatgaagacgagg)	9430	601 bp	Exon 4

Abbreviations: bp = base pair; (q)PCR = (quantitative) polymerase chain reaction.

**Table 7:** Overview of the RT-(q)PCR parameters published. A red cross signifies that no information was provided; a green check mark means information was provided. RG = reference gene (e.g., GAPDH).

Publication Parameter	Baldion et al. (2021) [8]	Feng et al. (2021) [15]	Baldion et al. (2021) [9]	Sabandal et al. (2020) [11]	Liu et al. (2020) [14]	Meng et al. (2019) [13]	Wen et al. (2017) [12]	Sun et al. (2018) [12]
RT-PCR	✓	✓	✓	✓	✓	✓	✓	✓
RNA quality	✗	✗	✗	✗	✗	✗	✗	✗
cDNA priming	✗	✗	oligo-dT or random primers	✗	✗	✗	✗	✗
PCR primers sequence	✓	✓	✓	✓	✓	✓	✓	✓
Accession number provided	(✗)	✗	✓	✗	✗	✗	✗	✗
Temperature/volume/time	✓/✓/✓	✗/✗/✗	✓/✗/✓	✓/✗/✗	✓/✗/✓	✗/✗/✗	✓/✗/✓	✓/✗/✓
RNA/water control	✗/✗	✗/✗	✗/✗	✗/✗	✗/✗	✗/✗	✗/✗	✗/✗
PCR efficiency	LinRegPCR	✗	LinRegPCR	no qPCR	✗	✗	REST 2005	no qPCR
Normalization	single, validated RG	single, unvalidated RG	single, validated RG		single, unvalidated RG	single, unvalidated RG	single, validated RG	
Biological replicates	✗	✗	✗	✗	✗	✗	✗	✗

## Data Availability

Not applicable.
